# The PICALM Protein Plays a Key Role in Iron Homeostasis and Cell Proliferation

**DOI:** 10.1371/journal.pone.0044252

**Published:** 2012-08-30

**Authors:** Paula B. Scotland, Jessica L. Heath, Amanda E. Conway, Natasha B. Porter, Michael B. Armstrong, Jennifer A. Walker, Mitchell L. Klebig, Catherine P. Lavau, Daniel S. Wechsler

**Affiliations:** 1 Division of Pediatric Hematology-Oncology, Duke University Medical Center, Durham, North Carolina, United States of America; 2 Department of Pharmacology and Cancer Biology, Duke University Medical Center, Durham, North Carolina, United States of America; 3 Department of Laboratory Medicine and Pathology, Mayo Clinic, Rochester, Minnesota, United States of America; Wayne State University, United States of America

## Abstract

The ubiquitously expressed phosphatidylinositol binding clathrin assembly (PICALM) protein associates with the plasma membrane, binds clathrin, and plays a role in clathrin-mediated endocytosis. Alterations of the human *PICALM* gene are present in aggressive hematopoietic malignancies, and genome-wide association studies have recently linked the *PICALM* locus to late-onset Alzheimer's disease. Inactivating and hypomorphic *Picalm* mutations in mice cause different degrees of severity of anemia, abnormal iron metabolism, growth retardation and shortened lifespan. To understand PICALM’s function, we studied the consequences of PICALM overexpression and characterized PICALM-deficient cells derived from mutant *fit1* mice. Our results identify a role for PICALM in transferrin receptor (TfR) internalization and demonstrate that the C-terminal PICALM residues are critical for its association with clathrin and for the inhibitory effect of PICALM overexpression on TfR internalization. Murine embryonic fibroblasts (MEFs) that are deficient in PICALM display several characteristics of iron deficiency (increased surface TfR expression, decreased intracellular iron levels, and reduced cellular proliferation), all of which are rescued by retroviral *PICALM* expression. The proliferation defect of cells that lack PICALM results, at least in part, from insufficient iron uptake, since it can be corrected by iron supplementation. Moreover, PICALM-deficient cells are particularly sensitive to iron chelation. Taken together, these data reveal that PICALM plays a critical role in iron homeostasis, and offer new perspectives into the pathogenesis of PICALM-associated diseases.

## Introduction

The *PICALM* (phosphatidylinositol binding clathrin assembly protein) gene was originally identified as a translocation partner for *AF10 (MLLT10)* in the U937 leukemic cell line [1]. The ubiquitously expressed PICALM protein is involved in clathrin-mediated endocytosis [1]–[4]. PICALM localizes to developing clathrin-coated vesicles on the cytoplasmic side of the plasma membrane, associates with components of the endocytic machinery, and is required for endocytosis [2], [5]. Previous studies have demonstrated that overexpression of PICALM inhibits endocytosis of both the Transferrin (Tf) Receptor (TfR) [2] and Epidermal Growth Factor Receptor (EGFR) [2], [6]. Knockdown studies have shown that the absence of PICALM results in enlarged and abnormally shaped endocytic vesicles [4]. PICALM deficiency, as seen in mice homozygous or hemizygous for *Picalm* ^fit1^ ENU (N-ethyl N-nitrosourea)-induced alleles [7], results in impaired hematopoiesis, anemia, abnormal iron metabolism, growth defects, and shortened life span [8]–[11]. In addition to its role in endocytosis, PICALM has been implicated in transcriptional regulation, and like several other proteins involved in the endocytosis machinery (EPS15, EPN1 and α-adaptin), transiently localizes to the nucleus [12], [13]. However, PICALM’s role in the nucleus is poorly understood.

The structural and functional domains of PICALM are similar to those of the neuronal specific protein AP180. These proteins are highly homologous but are differentially expressed. AP180 is only expressed in neurons, primarily in axons, while PICALM appears to be ubiquitous [14]–[18]. Both proteins have an amino-terminal ENTH (Epsin N-Terminal Homology) domain responsible for membrane association with PIP_2_ and a carboxy-terminal region that is believed to be involved in clathrin association [19]–[22]. Both proteins mediate clathrin assembly at the site of endocytic vesicle invagination and are thought to participate in controlling vesicle size [4].

The human *PICALM* gene (located at chromosomal band 11q14) has been implicated in several clinical disorders. In leukemias and lymphomas, *PICALM* has been identified as a translocation partner for the *AF10* transcription factor gene (10p12) [1], [3], [23], and also for the Mixed Lineage Leukemia (*MLL*) histone methyltransferase gene (11q23) [24]. The resulting fusion genes, *PICALM-AF10* and *MLL-PICALM*, have been found in aggressive hematologic malignancies [25]–[27]. While the chromosomal translocation that gives rise to *MLL-PICALM* is very rare [24], the *PICALM-AF10* translocation is found in various hematopoietic malignancies and notably in 5–10% of T-cell acute lymphoblastic leukemias [3], [26]. Although the oncogenic properties of *PICALM-AF10* have been characterized [3], [28], the specific role of *PICALM* in leukemogenesis has not been investigated. *PICALM* has also recently been implicated in late-onset Alzheimer's disease by genome-wide association studies [29]. It had been previously hypothesized that faulty endocytosis plays a role in the neuronal degeneration associated with Alzheimer's disease [30], and this notion is supported by the detection of specific *PICALM* SNPs in Alzheimer’s Disease patients [29], [31]–[33].

The present studies explore the physiological role of PICALM by determining the effects of *PICALM* overexpression in HEK293 cells and loss of PICALM expression in mouse embryonic fibroblasts (MEFs) derived from PICALM-deficient (*Picalm* ^fit1–5R^/*Picalm* ^fit1–5R^, hereafter referred to as *Picalm* ^NULL^) mice [7]. Overexpression of PICALM impairs TfR endocytosis, and mutagenesis of the *PICALM* cDNA has allowed us to identify the domains that are critical for this function. Studies of PICALM-deficient cells corroborated the essential role of PICALM in endocytosis and also revealed a previously unappreciated role in iron homeostasis. The rate of TfR endocytosis, the expression of total TfR protein and mRNA, and intracellular iron levels were all affected in PICALM-deficient cells. These cells also display a proliferation defect compared with their wild type (WT) counterparts. Rescue of PICALM expression by retroviral transduction restores endocytosis, TfR expression, intracellular iron levels, and proliferation in these cells. Furthermore, the proliferation defect of PICALM-deficient cells appears to be due to iron deficiency, as iron supplementation restores their proliferation to normal levels. Finally, PICALM-deficient cells are more sensitive to the growth inhibitory effect of iron chelation, further illustrating the importance of PICALM in intracellular iron homeostasis. Our findings establish that PICALM is required for endocytosis of the TfR, and that this in turn impacts the ability of cells to import iron. This study is also the first to demonstrate a role for PICALM in cellular proliferation. These observations raise the possibility that perturbed iron metabolism may play a role in *PICALM*-associated disease states.

## Results

### A PICALM Carboxy-terminal Domain is Essential for Inhibition of Endocytosis and Mediates Clathrin Binding

**Figure 1:**
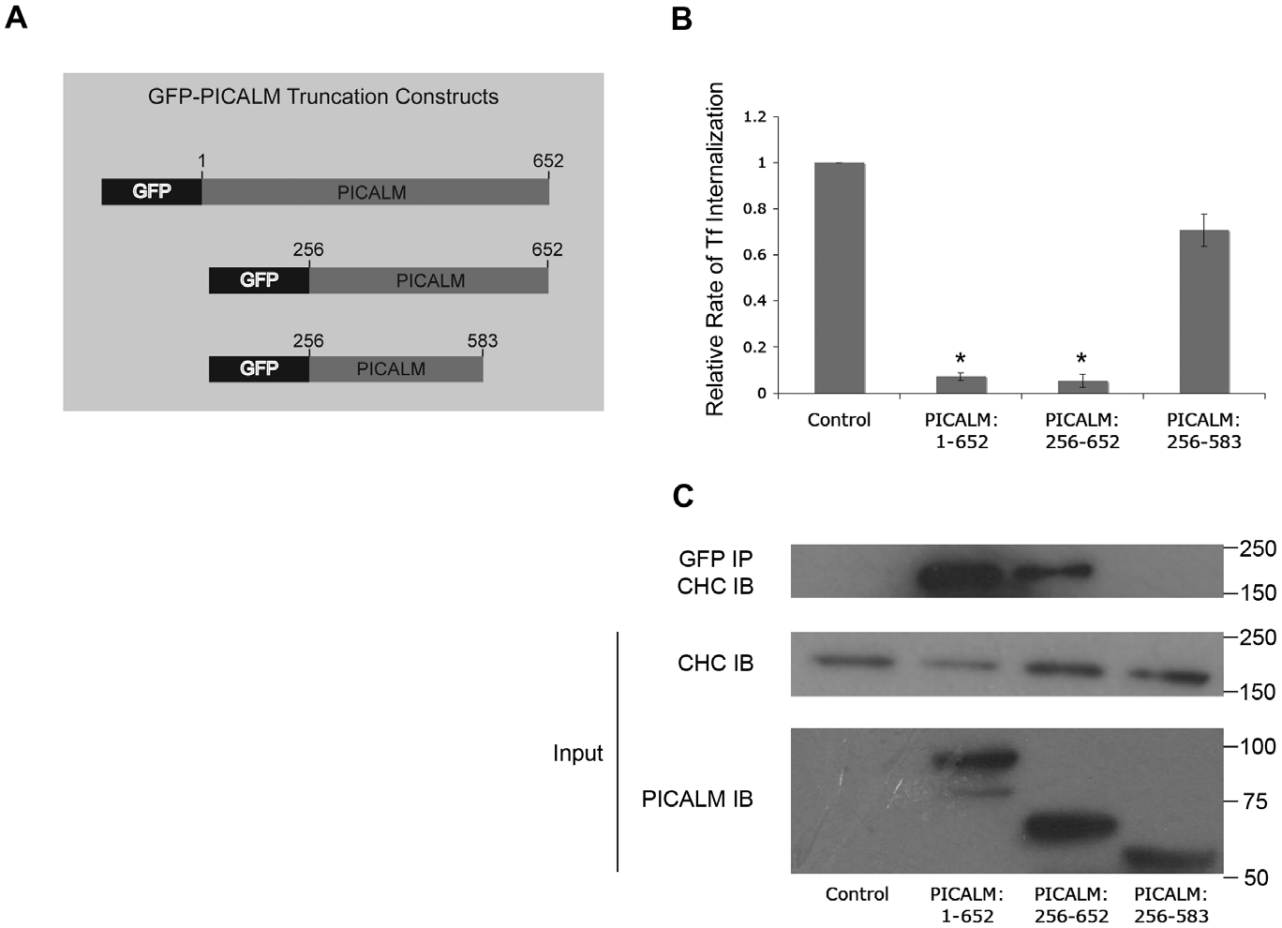
The carboxy-terminal 69 amino acids of PICALM are essential for binding clathrin and interfering with TfR endocytosis. (**A**) Schematic representation of amino-terminal-tagged GFP-PICALM fusion proteins. (**B**) TfR internalization in HEK293 cells transiently transfected with empty vector (control), full length PICALM (PICALM: 1–652), amino-terminal truncated PICALM (PICALM: 256–652), or a PICALM mutant missing both the amino and carboxy-terminal regions (PICALM: 256–583). The relative rate of endocytosis was calculated as described in Materials and Methods. N_exp_  = 11. *p<0.001 compared with control. (**C**) Western blot of proteins co-immunoprecipitated (IP) with anti-GFP antibody and immunoblotted (IB) with anti-clathrin heavy chain (CHC) antibody (upper panel). Cell lysates (input) were also immunoblotted with anti-CHC and anti-PICALM antibodies (lower panels). The molecular weights of GFP-PICALM, GFP-PICALM:256–652 and GFP-PICALM:256–583 are 97kD, 70kD and 62 kD, respectively.

Previous studies have demonstrated that overexpression of full-length PICALM impairs endocytosis [2], likely by a dominant negative mechanism whereby higher levels of PICALM sequester components of the endocytic machinery. To determine which specific PICALM regions are required in endocytosis, we prepared GFP-labeled PICALM truncation mutants missing the amino- and carboxy-terminal domains (**Figure 1A**). As expected, the rate of TfR internalization in transfected HEK293 cells overexpressing GFP-PICALM was reduced by >90% in comparison with cells transfected with empty GFP vector (**Figure 1B**, GFP vs. PICALM:1–652). Although the amino-terminal 255 amino acid residues of PICALM (PICALM:256–652) alone are not necessary for this inhibitory effect, a deletion mutant truncated at amino acid 583 (PICALM:256–583) resulted in a significant loss of the ability to inhibit endocytosis (**Figure 1B** and **Figure S1A**). These results indicate that the carboxy-terminal 69 amino acid (aa) residues of PICALM play an important role in clathrin-mediated endocytosis (CME).

To determine whether an interaction with clathrin correlates with PICALM-mediated inhibition of endocytosis, full-length and truncated GFP-PICALM proteins were immunoprecipitated from transfected cells (anti-GFP antibody) and probed with an anti-clathrin heavy chain antibody. The same GFP-PICALM proteins that inhibit TfR internalization, full-length (aa 1–652) and amino-terminally truncated PICALM (aa 256–652), also bind to and immunoprecipitate clathrin (**Figure 1B & C**). In contrast, a mutant protein lacking both the C-terminal 69 amino acids and the N-terminal 255 residues of PICALM (aa 256–583), which failed to inhibit TfR internalization, does not interact with clathrin (**Figure 1B & C**, and **Figure S1A,** lower blot). These observations suggest that the interaction of PICALM with clathrin via its C-terminal 69 aa residues could be a mechanism for its inhibitory effect on CME when it is overexpressed in HEK293 cells.

To identify the specific amino acids within the PICALM carboxy-terminal region that are responsible for CME inhibition and clathrin binding, a series of deletion and point mutations were analyzed. These PICALM variants were expressed in HEK293 cells, and the rate of TfR internalization was measured. Analysis of the deletion mutants revealed that inhibition of TfR internalization increased progressively as more of the PICALM carboxy-terminal region was present (**Figure S1A top**). In addition, only truncation mutants that inhibited TfR internalization also co-immunoprecipitated with clathrin heavy chain (**Figure S1A bottom**), further supporting the importance of clathrin binding in PICALM’s role in endocytosis. PICALM overexpression may act through a dominant negative mechanism by sequestering clathrin. To identify the critical residues of PICALM that are required for inhibition of CME, site-directed mutagenesis spanning amino acids 583 to 652 was performed using NAAIRS mutagenesis to minimize changes in protein structure [34]. NAAIRS mutagenesis involves substituting nucleotides encoding six sequential amino acids of interest with those encoding amino acids asparagiNe-Alanine-Alanine-Isoleucine-aRginine-Serine (NAAIRS). This sequence is capable of forming either an alpha helix or a beta-sheet, and thus is more suitable for maintaining the secondary and tertiary structure of the protein while testing the function of the specific amino acids of interest. Each of eleven distinct NAAIRS mutants (**Figure S1C**) inhibited TfR internalization, much like full-length, normal PICALM (**Figure S1B top**). In addition, each of these mutants also co-immunoprecipitated with clathrin, albeit to varying degrees (**Figure S1B bottom**). In fact, one double mutant, NPF-IGYGIP, chosen for further study because it bound significantly less clathrin, was still able to inhibit endocytosis. Because extensive point mutagenesis of the *PICALM* gene within the last carboxy-terminal 69 amino acids did not identify a single specific site responsible for clathrin association and inhibition of TfR internalization, it is likely that there are multiple cooperating motifs within this region that ensure association with clathrin and possibly other components of the clathrin-mediated endocytic machinery**.**

### PICALM-deficient MEFs Display Increased Surface TfR and Reduced Endocytosis

**Figure 2:**
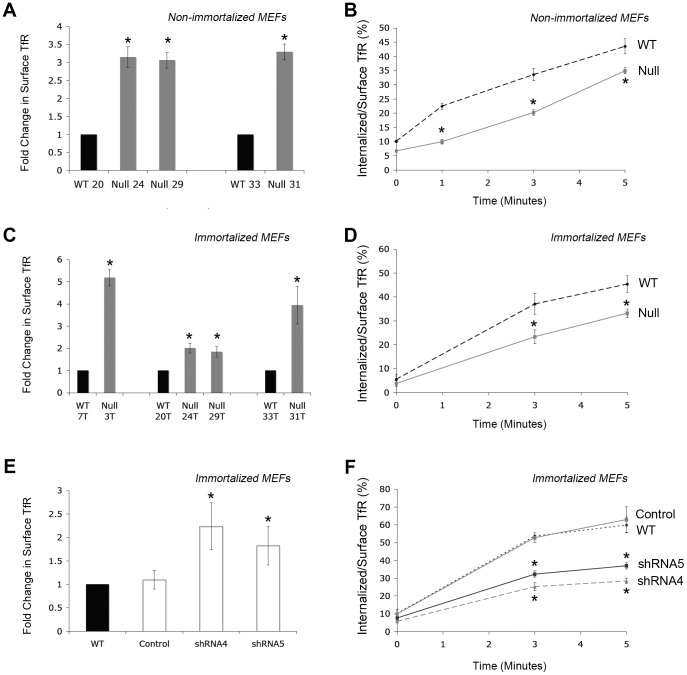
Absence of PICALM results in a decreased rate of internalization and increased cell surface expression of TfR in MEFs. The level of cell surface TfR expression normalized to levels in WT cells (**A,C,E**) and the rate of TfR internalization (**B,D,F**) was measured in non-immortalized MEFs (**A,B**), immortalized MEFs (**C,D**), or PICALM shRNA knockdown WT (line 7T) MEFs (**E,F**). (**A**) Surface TfR expression in non-immortalized WT and *Picalm* ^NULL^ MEFs. N_exp_  = 4. *p<0.02 compared with WT. (**B**) TfR internalization at three different time points in independently derived non-immortalized WT (lines 20, 33) and *Picalm* ^NULL^ (lines 24, 29, 31). N_exp_  = 2. *p<0.04 compared with WT. (**C**) Surface TfR expression in immortalized WT and *Picalm* ^NULL^ MEFs. N_exp_  = 4. *p<0.04 compared with WT. (**D**) TfR internalization in independently derived immortalized WT (lines 20T, 33T) and *Picalm* ^NULL^ (lines 24T, 29T, 31T). N_exp_  = 7. *p<0.01 compared with WT. (**E**) Surface TfR expression in shRNA transduced WT MEFs. N_exp_  = 4. *p<0.02 compared with WT. (**F**) TfR internalization in immortalized WT (line 7T) transduced with two separate PICALM shRNA retroviruses (shRNA4, shRNA5) or a control shRNA retrovirus. N_exp_  = 3. *p<0.01 compared with WT. In all cases, error bars denote standard error. Two-tailed Student’s t-test was used to compare means for TfR internalization (A,C,E).

Overexpression studies in HEK293 cells showed that excess levels of PICALM perturb TfR internalization, consistent with previous studies [2]. Interestingly, loss of PICALM function has also been reported to impair CME and receptor internalization [4]. To analyze the effects of PICALM deficiency and the requirement for PICALM in TfR endocytosis, we isolated embryonic fibroblasts from either normal or PICALM-deficient (*Picalm* ^fit1–5R^/*Picalm* ^fit1–5R^) mice. The ENU-induced *Picalm* ^fit1–5R^ allele has a point mutation in the splice donor site immediately downstream of *Picalm* exon 4 that causes aberrant splicing and encodes a severely truncated PICALM protein that includes only the first 116 amino acids [7]. Because the mutant protein lacks the putative clathrin-binding domain and almost all other known domains, the *Picalm* ^fit1–5R^ allele is considered to be a null [7]. Heterozygous mice were bred, and *Picalm^+/+^* or *Picalm^fit1^* ^–*5R/fit1*–*5R*^ (hereafter referred to as WT or *Picalm* ^NULL^, respectively) mouse embryonic fibroblasts (MEF) were isolated from E14 embryos. The MEFs were either grown as primary cells or immortalized with SV40 T/t antigen. Seven different immortalized lines (three WT lines, four *Picalm* ^NULL^ lines) were generated from three different pregnancies (**Figure S2A**). In all experiments, *Picalm* ^NULL^ cells were compared with WT cells derived from littermate embryos. Flow cytometry analysis of both primary and immortalized MEFs revealed that PICALM-deficiency was accompanied by a dramatic increase in surface TfR expression (2–5 fold increase compared to WT cells, **Figure 2A and 2C**). We used a flow cytometry-based assay to measure endocytosis of TfR over time and because of the considerable difference in expression levels between PICALM^NULL^ and WT cells, TfR uptake was normalized to the total amount of TfR at the cell surface. At 3 minutes, 20±1 or 23±3 (percent±SE) of TfR was internalized in non-immortalized or immortalized PICALM-deficient MEFs, respectively, while 33±3 or 37±5% of TfR was internalized in their WT counterparts (**Figure 2B and 2D**). Remarkably, despite the reduced efficiency of TfR internalization in PICALM-deficient MEFs, the increased cell surface levels of the receptor resulted in a higher net amount of internalized TfR in PICALM deficient cells (4,800±200 and 24,300±400, MFI±SE, non-immortalized or immortalized MEFs, respectively) compared with WT control MEFs (2,750±900 and 15,300±3,400) (raw data before normalization, not shown).

To confirm the role of PICALM in TfR endocytosis, we stably knocked down its expression using shRNAs into immortalized WT MEFs and also HEK293 cells. Two shRNAs (shRNA4 and shRNA5) that induced greater than 60% reduction of PICALM protein expression in MEFs were compared with a control shRNA (**Figure S2B**). Consistent with the phenotype of *Picalm* ^NULL^ MEFs, shRNA knockdown of *Picalm* expression was accompanied by an increase in surface TfR expression (**Figure 2E**) and a significant reduction of TfR internalization efficiency (**Figure 2F**). Similarly, knockdown of *Picalm* in HEK293 cells (**Figure S2C**) resulted in increased surface TfR (**Figure S2D**) and reduced TfR internalization efficiency (**Figure S2E**), confirming the observations in MEFs.

**Figure 3:**
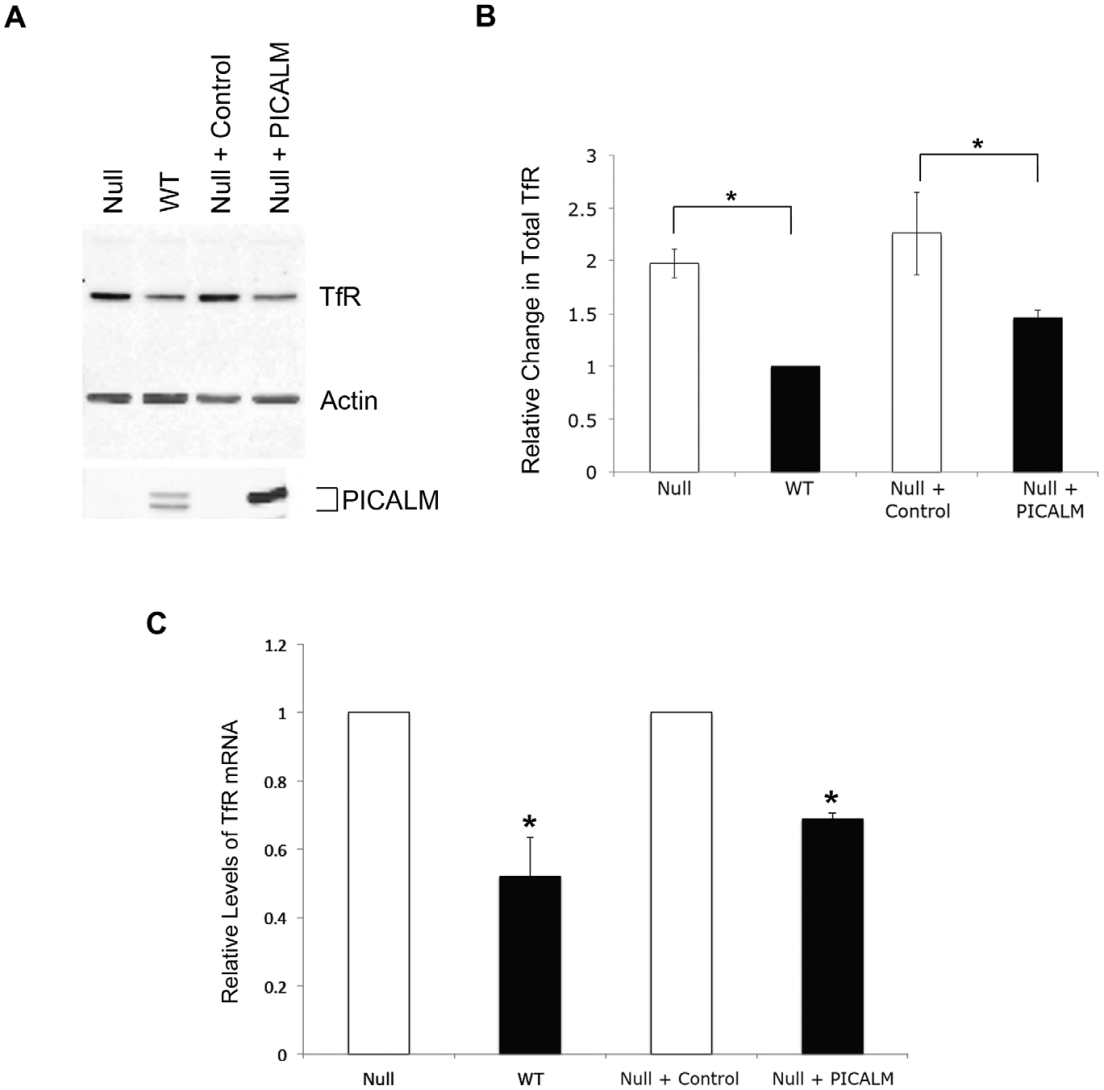
*PICALM-deficient* cells display increased total TfR protein and mRNA. (**A**) Immunoblot of total TfR protein, PICALM, and β-actin from *Picalm* ^NULL^ (Null), WT, *Picalm* ^NULL^ control MEFs infected with empty vector (Null + control), or *Picalm* ^NULL^ MEFs rescued with PICALM (Null + PICALM). *PICALM* cDNA encodes for the larger isoform. (**B**) Quantitation of TfR shown in panel A immunoblot normalized to β-actin, with values shown relative to TfR level in WT MEFs. N_exp_  = 3. *p<0.04. (**C**) RT-PCR quantitation of TfR mRNA in *PICALM-deficient* (Null) MEFs, WT MEFs, *Picalm* ^NULL^ MEFs transduced with empty vector (Null + Control) or rescued with PICALM (Null + PICALM). Results are normalized to levels in Null MEFs or Null + Control cells. N_exp_  = 3. *p<0.04 compared with WT cells.

In order to determine whether the increase in surface TfR was caused by changes in TfR expression levels or mislocalization of the protein, total cell TfR protein was quantified by infrared fluorescence analysis of immunoblots. *Picalm* ^NULL^ MEFs showed an approximately two-fold TfR increase compared with WT controls (**Figure 3A and 3B**). Transcript quantification by RT-PCR analysis similarly showed higher levels of TfR mRNA in *Picalm* ^NULL^ cells (**Figure 3C**). The increased expression of TfR in PICALM-deficient cells likely reflects a state of relative iron deficiency, characterized by increased TfR mRNA stability and protein translation, and is consistent with the iron deficiency phenotype originally described in the *fit1* mutant mice [7], [8].

### The PICALM Carboxy-terminal Region is Essential for Rescue of the PICALM-deficient Phenotype

**Figure 4:**
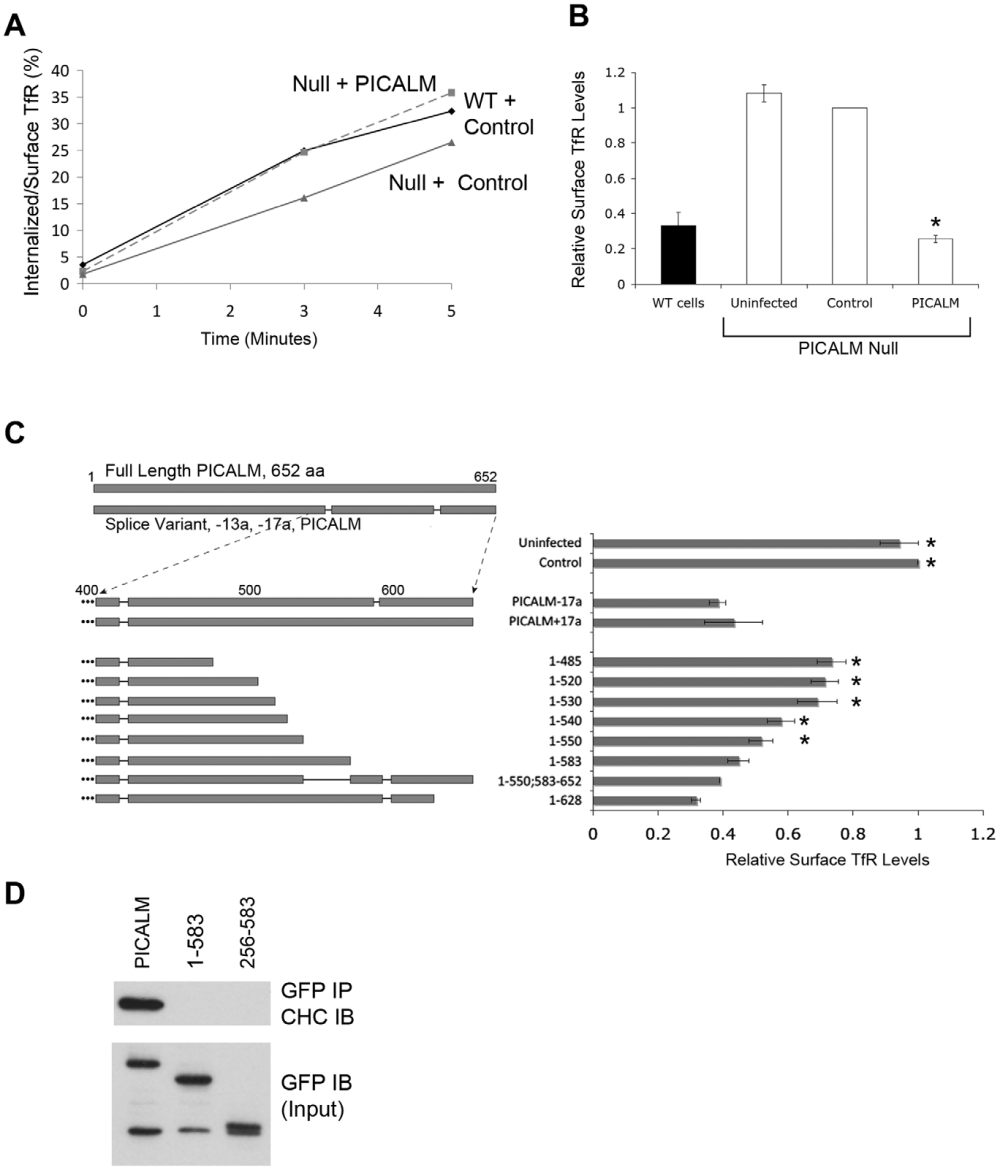
PICALM expression rescues the PICALM-deficient phenotype, and the PICALM carboxy-terminal domain plays an essential role. (**A**) Representative experiment showing rate of TfR internalization in *Picalm* ^NULL^ MEFs (line 31T) stably infected with PICALM or empty vector (control) compared with WT (line 33T) MEFs. (**B**) Surface TfR expression in WT MEFs (7T, 20T, 33T) or *Picalm* ^NULL^ MEFs (lines 3T, 24T, 29T, 31T) that were uninfected, stably infected with empty vector (control) or PICALM. Surface TfR levels were normalized to levels in control *Picalm* ^NULL^ MEFs. N_exp_  = 8. *p<0.001 compared with control. TfR endocytosis and surface TfR levels measured in three independent *Picalm* ^NULL^ lines is shown in Figure S3. (**C**) Deletion mutagenesis of the PICALM carboxy-terminal domain (constructs depicted in left panel) demonstrates that the ability to rescue surface TfR expression is dependent on the PICALM carboxy-terminal domain. N_exp_  = 3. *p<0.02 compared with PICALM-17a. (**D**) Co-immunoprecipitation of lysates from HEK293 cells transiently transfected with full length PICALM (PICALM: 1–652), carboxy-terminal truncated PICALM (PICALM: 1–583), or a PICALM mutant missing both the amino and carboxy-terminal regions (PICALM: 256–583). Extracts were immunoprecipitated (IP) with anti-GFP antibody and immunoblotted (IB) with anti-clathrin heavy chain (CHC) antibody (upper panel). Cell lysates (input) were also immunoblotted with anti-PICALM antibodies (lower panel).

To confirm that the phenotype of *Picalm* ^NULL^ MEFs results from the absence of PICALM, we used retroviral transduction to restore PICALM expression. Immortalized PICALM-deficient MEF lines were stably infected with either an empty retroviral vector or a vector encoding full length PICALM. As shown in **Figure 4A**, expression of full-length PICALM rescued the efficiency of TfR internalization. Expression of PICALM also lowered cell surface expression of TfR protein in the different MEF lines (**Figure 4B**). Furthermore, both total TfR protein and mRNA levels decreased upon PICALM transduction of *Picalm* ^NULL^ cells (**Figures 3B and 3D**). The ability of PICALM to rescue the efficiency of TfR endocytosis and to reduce its surface expression were confirmed in three additional independently derived *Picalm* ^NULL^ MEF lines (Figures **S3A**, **S3B).** These findings demonstrate that PICALM deficiency is responsible for defective TfR endocytosis and altered TfR expression in murine cells.

We used the PICALM rescue assay of *Picalm* ^NULL^ cells to determine the regions of the protein required to lower surface expression of TfR. To this end, a series of PICALM deletion/truncation mutants was stably expressed in the 3T *Picalm* ^NULL^ line, and surface TfR levels were analyzed by flow cytometry. While full length PICALM reduced the levels of surface TfR more than two-fold, constructs lacking the carboxy-terminal 122 amino acids (1–485, 1–520, 1–530), were significantly less potent (**Figure 4C**). The progressive inclusion of residues 540 to 583 (1–540, 1–550, 1–583) increased the ability to rescue the PICALM-deficient phenotype. In particular, the 1–583 truncation mutant was able to rescue the surface TfR overexpression phenotype to the same extent as normal PICALM (**Figure 4C**), even though it lacks the C-terminal domain important for clathrin interaction (**Figure 1C** **and** **4D**). These results indicate that the N-terminal 583 aa of PICALM are critical for rescuing surface TfR overexpression, and that the 530–583 domain is of particular importance for this rescue.

Little is known about the functional significance of alternatively spliced PICALM variants. Given the carboxy-terminal position of the eight amino acids (NGMHFPQY, at position 593) encoded by exon 17a, we tested the impact of their inclusion on PICALM's ability to restore surface TfR levels in *Picalm* ^NULL^ cells. The presence of this exon did not affect PICALM’s activity (**Figure 4C**, PICALM-17a vs. PICALM+17a). Likewise, the inclusion of alternatively spliced exon 13a did not alter the ability of PICALM to rescue TfR expression (data not shown). The functional role of these alternatively spliced variants remains to be discovered.

### *Picalm* ^NULL^ Cells are Iron Deficient

**Figure 5:**
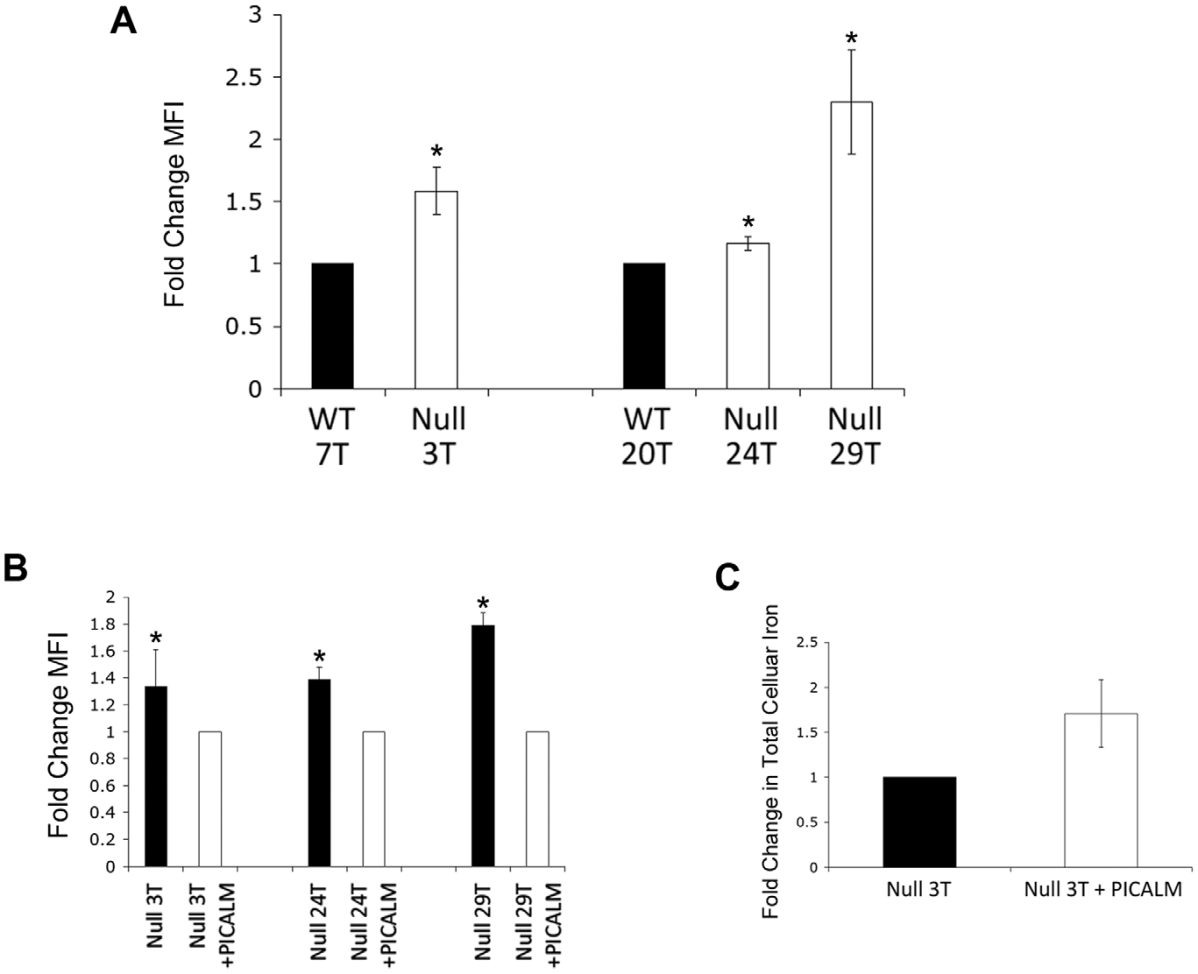
*PICALM-deficient* MEFs exhibit decreased intracellular iron compared with WT or PICALM rescued MEFs. (**A**) Chelation of Phen Green SK (the fluorescence of which inversely correlates with intracellular iron levels) was measured in WT (lines 7T, 20T) or *PICALM-deficient* (*Picalm* ^NULL^ – lines 3T, 24T, 29T) MEFs. Values were normalized to those in WT MEFs. N_exp_  = 3. *p<0.04 compared with WT. (**B**) Chelation of Phen Green SK in *Picalm* ^NULL^ cells that were uninfected or rescued with PICALM. Values are normalized to levels in PICALM-rescued cells. N_exp_  = 3. *p<0.04 compared with PICALM-rescued cells. (**C**) Total intracellular iron levels in *Picalm* ^NULL^ (line 3T) and PICALM rescued MEFs, normalized to levels in *Picalm* ^NULL^ cells. N_exp_  = 3. MFI: mean fluorescence intensity.

Tf plays an essential role in importing iron into cells by binding to its receptor, TfR, on the cell surface, followed by clathrin-mediated endocytosis. To determine whether iron homeostasis is perturbed in *Picalm* ^NULL^ cells, we quantified levels of intracellular iron. We first measured the labile iron pool using the iron chelating reagent Phen Green SK (PGSK) [35]. PGSK is a membrane permeable dye, the fluorescence of which is quenched in the presence of labile iron (Fe^2+^ or Fe^3+^). Thus, the fluorescence of PGSK is inversely proportional to the amount of intracellular labile iron. In the 3T, 24T and 29T *Picalm* ^NULL^ lines, the PGSK fluorescence was higher than in the WT counterparts, indicating that the labile iron pool of *Picalm* ^NULL^ cells was reduced (**Figure 5A**). To limit the intrinsic variability between independent cell lines, we compared PGSK fluorescence in *Picalm* ^NULL^ lines with or without PICALM rescue. Retroviral transduction of PICALM in three separate *Picalm* ^NULL^ cell lines (3 T, 24 T, and 29 T) significantly increased PGSK quenching (**Figure 5B**), indicating that PICALM expression restored intracellular labile iron pool levels. As an alternative approach, we used mass spectroscopy to measure whole cell iron content. Total iron levels were measured in the *Picalm* ^NULL^ line 3 T transduced with either control vector or PICALM (**Figure 5C**). Although the difference did not reach statistical significance, PICALM rescued cells contained more iron than vector controls, further suggesting a role of PICALM in increasing cellular iron.

### Insufficient Intracellular Iron Limits the Proliferation of *Picalm* ^NULL^ Cells

**Figure 6:**
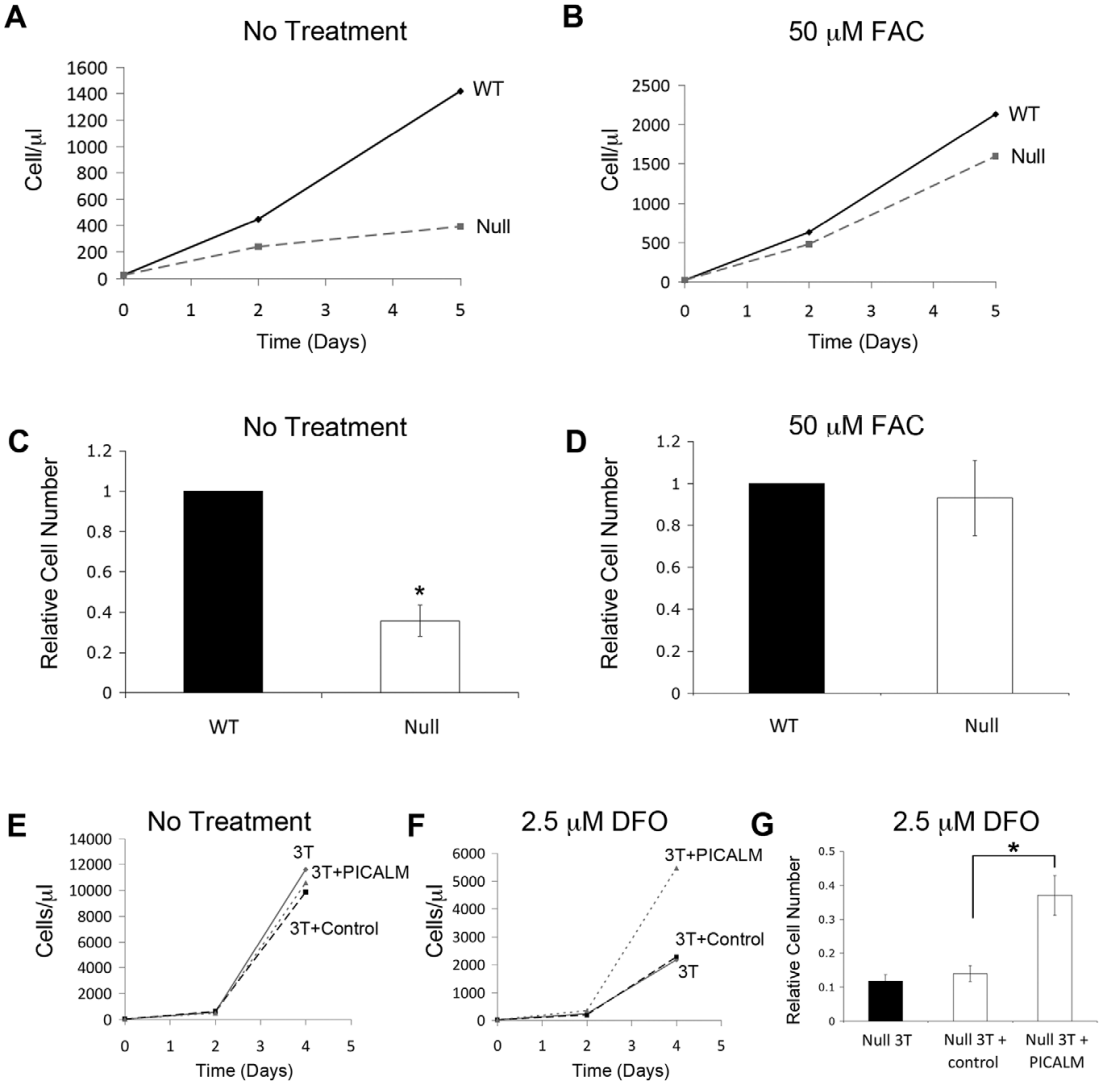
*PICALM-deficient* MEFs proliferate more rapidly in the presence of iron supplementation and are more sensitive to iron chelation. (**A,B**) Representative proliferation curves of non-immortalized *Picalm* ^NULL^ and WT MEFs without (A) and with (B) 50 µM ferric ammonium citrate (FAC). FAC restores proliferation in *Picalm* ^NULL^ cells to levels in WT cells. (**C,D**) Mean cell numbers (+/− standard error) of non-immortalized *Picalm* ^NULL^ and WT MEFs grown for 5 days without (C) and with (D) 50 µM FAC. N_exp_  = 3. *p<0.04 compared with WT cells. (**E,F**) Representative proliferation curves of immortalized *Picalm* ^NULL^ MEFs transduced with empty vector (control) or *PICALM* grown for 4 days in the absence (E) or presence (F) of 2.5 µM deferoxamine (DFO). (**G**) Mean cell number of *Picalm* ^NULL^ MEFs untransduced (Null 3T) or transduced with empty vector (control) or PICALM after 4 days of culture in the presence of 2.5 µM DFO treatment relative to number of untreated cells. N_exp_  = 4. *p<0.003 compared with PICALM rescued cells.

While establishing MEF cultures from *Picalm* ^NULL^ and WT embryos, we observed that PICALM-deficient MEFs consistently took longer to reach confluence. To determine whether PICALM influences the rate of cell proliferation, we plated non-immortalized *Picalm* ^NULL^ and WT MEFs at fixed densities and compared their proliferation rates over 5 days (**Figure 6A, 6C**). WT MEFs proliferated at considerably higher rates than *Picalm* ^NULL^ cells (the cell expansion at day 5 was on average 2.5-fold higher for WT compared with *Picalm* ^NULL^ MEFs). To determine whether the reduced proliferation of *Picalm* ^NULL^ MEFs was attributable to lower levels of intracellular iron, proliferation assays were repeated in the presence of supplemental iron in the form of ferric ammonium citrate (FAC). FAC is a low-molecular-weight form of iron that can penetrate into cells by bypassing the transferrin endocytic pathway [36]. When FAC (50 µM) was added to culture medium, the proliferation rate of PICALM-deficient MEFs was restored to that of WT cells (**Figure 6B, 6D**). This demonstrates that the growth of PICALM-deficient cells is limited by iron availability and further supports the importance of PICALM in ensuring optimal iron import into cells.

Finally, we examined whether *Picalm* ^NULL^ MEFs are more sensitive to a reduction in extracellular iron concentration. To this end, the proliferation of immortalized MEFs was measured in the presence of the iron-chelating drug deferoxamine (DFO). While immortalized *Picalm* ^NULL^ MEFs displayed a similar high rate of proliferation in regular culture media regardless of the presence of *PICALM* (**Figure 6E**), *Picalm* ^NULL^ cells transduced with empty vector were more sensitive to the growth inhibitory effect of DFO than *Picalm* ^NULL^ cells transduced with PICALM (**Figure 6F, 6G**). These findings indicate that when iron concentration is limiting, the presence of PICALM is required for optimal cell proliferation.

## Discussion

Internalization of transferrin-bound iron via the transferrin receptor (TfR) is the primary mechanism of cellular iron import, and is dependent on clathrin-mediated endocytosis (CME). A disruption of CME would therefore be expected to reduce intracellular iron levels. PICALM, a homologue of the previously identified endocytic protein AP180 [1], is involved in promoting clathrin coat assembly in the early stages of vesicle budding from the membrane [2], [6], [37]. Overexpression of PICALM impairs endocytosis of both the TfR and the Epidermal Growth Factor Receptor (EGFR), likely via dominant negative interference [2], [6]. Previous studies using siRNAs to downregulate PICALM expression in HeLa and HEK293 cells concluded that PICALM is not required for TfR endocytosis [4], [6], [38], [39]. However, depletion of PICALM impairs endocytosis of EGFR and VAMP2 (synaptobrevin 2) [6], [38], in addition to causing defects in the size and shape of clathrin-coated structures [4], and affecting the maturation of clathrin-coated pits [5]. Furthermore, a role for PICALM in sorting of R-SNAREs (VAMP2, VAMP3, VAMP8) into endocytic vesicles has recently been described [39].

An indication of a critical role for PICALM in iron metabolism came from studies of the *fit1* mutant mouse. *fit1* mice were initially generated using N-ethyl-N-nitrosourea (ENU) mutagenesis, and were subsequently shown to have a nonsense point mutation in the *Picalm* gene resulting in a truncated and nonfunctional PICALM protein [7]. Mice homozygous or hemizygous for *Picalm* ^fit1^ mutations display dramatic growth retardation compared with their normal littermates [8], [40]. These mice also show a distinct hematologic phenotype: they are anemic and have hypochromic red blood cells. Serum and tissue iron levels are also significantly lower in all but liver cells, which show a dramatic increase in iron stores [10]. Here, we characterize TfR endocytosis and proliferation properties of Picalm-deficient MEFs derived from *fit1* mice. Our studies suggest a defect in iron homeostasis as a contributory mechanism for the phenotype of these mice.

Our results demonstrate that internalization of TfR is impaired in Picalm^NULL^ MEFs and shRNA treated MEFs. Importantly, a recent report [41] also found that Tf endocytosis was impaired in erythroid cells and MEFs derived from *PICALM*-deficient mice, corroborating our observations. It is possible that the discrepancy of our results and those of Suzuki et al. [41] with previous reports suggesting that PICALM knockdown does not affect TfR endocytosis [4], [6], [38], [39] relates to the use of different methodologies to assess endocytosis, such as length and temperature of incubations and inclusion of acid washes; notably, previous reports used either fluorescence microscopy [38] or isotope labeled Tf [6], [39] to quantitate TfR internalization, whereas we and Suzuki et al. [41] used flow cytometry. We also measured the fraction of TfR internalized relative to the total surface TfR expression, while other reports measured the absolute amount of TfR internalized. This is of importance because the relative reduction in TfR internalization seen in *PICALM*-deficient cells could be due to a saturation of the cellular endocytic machinery resulting from the overexpression of TfR secondary to iron deficiency. Alternatively, the complete absence of functional PICALM in *fit1^−/−^* MEFs might be expected to have a greater impact on TfR endocytosis than RNA interference-based knockdown approaches, which do not necessarily result in a complete absence of functional PICALM. To address this possibility, we used shRNA to knockdown PICALM expression in wild type MEFs and in HEK293 cells, and found that this also results in impaired TfR endocytosis (**Figure 2E, 2F, S** **2D, S2E**). Of note, we have also observed impaired TfR endocytosis in *Picalm* ^NULL^ murine hematopoietic cells (data not shown) suggesting that the requirement of PICALM for TfR endocytosis is not cell type specific. Thus, it seems most likely that the use of different assays for TfR internalization may be the reason for the the apparent discrepant results about the role of PICALM in TfR endocytosis that have been reported in the literature.

Consistent with a defect in TfR endocytosis, PICALM-deficient MEFs display features of iron deficiency, including increased TfR expression at the protein and transcript levels, reduced concentrations of intracellular iron, and a defect in proliferation that can be restored by iron supplementation. The expression of TfR is regulated post-transcriptionally by iron-responsive elements in the 3′ UTR that stabilize TfR mRNA when intracellular iron levels decrease [42]. Consistent with an iron deficient state, *Picalm* ^NULL^ MEFs display an approximately two-fold increase in both TfR mRNA and protein levels compared to WT cells (**Figure 3**). The defect in TfR internalization also likely contributes to the more pronounced two- to five-fold increase in cell surface TfR seen by flow cytometry in the *Picalm* ^NULL^ MEFs (**Figure 2D**). Although the TfR internalization defect may play a causal role in the iron deficiency of *Picalm* ^NULL^ cells, the compensatory increase in TfR expression appears to enable the cells to internalize iron in sufficient amounts. This raises the possibility that PICALM is required for other steps that mediate the release of iron from endocytic vesicles; this could relate to PICALM’s role in endocytosis of R-SNAREs that can be expected to perturb trafficking of various proteins through the endocytic pathway [39].

One of the strengths of the PICALM-deficient MEF system is the ability to rescue the mutant phenotype (increased surface TfR expression, impaired TfR endocytosis, decreased intracellular iron, reduced proliferation rate) through restoration of PICALM expression by retroviral transduction. Previous studies have demonstrated that the carboxy-terminal third of the PICALM protein (aa 414–652) binds the clathrin heavy chain (CHC) [2]. Our overexpression studies in HEK293 cells (which have normal PICALM function) further narrowed the region involved in CHC binding to a 69 amino acid domain at the PICALM C-terminus (aa 583–652). However, in Picalm-deficient MEFs, we found that while the PICALM aa 1–530 construct is unable to rescue TfR expression, PICALM 1–583 is sufficient to achieve the same level of TfR surface expression as is achieved with normal PICALM (**Figure 4C**). This suggests that the PICALM aa 530–583 domain is of particular importance for rescue, and that specific clathrin binding by the C-terminal 69 aa is not required. It is possible that PICALM interacts with clathrin through aa 530–583 in the context of murine cells; this remains to be demonstrated formally. In summary, the effect of PICALM construct overexpression in Picalm-deficient MEFs (**Figure 4C**) appears to be different from that in PICALM-sufficient HEK293 cells (**Figure 1B**). We have shown that approximately 120 carboxy-terminal PICALM residues play a role in PICALM-dependent CME: aa 583–652 are required for inhibiton of TfR internalization in HEK293 cells, and aa 530–583 are essential for rescue of the iron-deficient phenotype in *Picalm* ^NULL^ MEFs. It is likely that different mechanisms are used under these specific circumstances, with a dominant negative effect at play in HEK293 cells, and interactions with other components of the endocytic machinery in *Picalm* ^NULL^ MEFs.

While the PICALM C-terminal region confers the ability to bind clathrin, no characteristic structural motif has been identified within it. Surprisingly, using scanning site-specific NAAIRS mutagenesis, we were unable to further narrow down a specific clathrin-binding domain. Immunoprecipitation studies revealed that NAAIRS mutations blanketing the entire carboxy-terminal 69 aa domain, singly and in combination, did not abolish clathrin binding. This suggests that PICALM must interact with clathrin through multiple binding sites within this domain, reminiscent of the repetitive clathrin assembly motifs present in the AP180 protein [22]. Taken together, we conclude that the iron deficiency phenotype of *fit1* mice is attributable to PICALM deficiency, and we have identified the carboxy-terminus of PICALM as the essential region.

The effect of PICALM on iron import has important biological consequences, since reduced levels of intracellular iron appear to limit the rate of cellular proliferation. This was revealed by observing that the slower growth of non-immortalized *Picalm* ^NULL^ MEFs could be corrected by supplementing the cells with iron. The proliferation defect observed in the *Picalm* ^NULL^ MEFs is consistent with the smaller size of *fit1* mutant mice [8]. It is curious that upon immortalization by SV40 T/t antigen, *Picalm* ^NULL^ MEFs did not show the same growth disadvantage (**Figure 6E**), suggesting that oncogenic transformation may involve alterations in metabolic pathways that lessen the need for iron or trigger other compensatory elements. However, when immortalized cells were challenged with the iron-chelating agent DFO, PICALM-deficient cells demonstrated higher sensitivity, indicating that these cells are still limited in their ability to internalize iron.

The observation that normal cell proliferation appears to require PICALM is intriguing given the involvement of *PICALM* in chromosomal translocations in hematopoietic malignancies. It is conceivable that PICALM fusion proteins may enhance cell proliferation by boosting iron uptake. In the case of *PICALM-AF10* translocations, many of the resulting fusion proteins contain almost the entire PICALM protein (except for the last four carboxy-terminal amino acids [25], [43]); the PICALM-AF10 fusion protein may still function in endocytosis and enhance iron uptake. Alternatively, PICALM rearrangements may confer a growth advantage by impairing endocytosis, with consequent upregulation of the surface expression of growth factor receptors, leading to persistent signaling and enhanced proliferation [44]–[47]. Leukemias with *PICALM* translocations also have *PICALM* haploinsufficiency, which could manifest as a relative deficiency of PICALM protein expression. The resulting defective endocytosis could render PICALM-deficient leukemia cells relatively iron deficient, and therefore more sensitive to reduced levels of extracellular iron. This raises the possibility that iron chelation could be of benefit in the treatment of patients with *PICALM* haploinsufficient leukemias. Of note, iron chelators are being studied as novel therapeutic agents in many malignancies, including leukemias [48].

Finally, the role of PICALM in iron homeostasis may also be relevant to the pathogenesis of Alzheimer's Disease (AD). It has long been recognized that brain iron content is altered in AD [49]–[52]: accumulation of excess brain iron is associated with AD, and “iron mismanagement” has been postulated to contribute to AD development [50]. Recently, multiple studies have shown the genetic association of *PICALM* polymorphisms with late onset AD [29], [33], [53], [54], although the mechanism by which PICALM contributes to the disease is unknown [55]. In particular, it is uncertain whether *PICALM* polymorphisms are associated with increased or decreased PICALM activity. Our observation that modulation of PICALM expression affects intracellular iron levels suggests a possible mechanism by which *PICALM* gene alterations could play a role in AD pathogenesis, and further investigation of this pathway may be warranted.

In summary, we have demonstrated that PICALM expression levels affect TfR endocytosis and alter iron homeostasis. PICALM-deficient cells proliferate more slowly than their WT counterparts, and PICALM re-expression in these cells restores endocytosis, intracellular iron levels and proliferation. PICALM-deficient cells are affected by manipulation of extracellular iron levels and are particularly sensitive to iron chelation. These observations raise the possibility that perturbed iron metabolism may play a role in *PICALM*-associated disease states.

## Materials and Methods

### Ethics Statement

All *in vivo* and euthanasia procedures in this study were carried out in strict accordance with the recommendations in the Guide for the Care and Use of Laboratory Animals of the National Institutes of Health. The animal studies described here have been approved by the Duke University Institutional Animal Care & Use Committee (IACUC) (Protocol# A029-10-02). All efforts were made to minimize animal suffering.

### Cell Culture

HEK293 (ATCC) and retroviral packaging Plat-E cells [56] (a generous gift of T. Kitamura), were maintained in DMEM supplemented with 10% fetal bovine serum, penicillin, and streptomycin (Invitrogen, Carlsbad, CA, USA). Mouse embryonic fibroblasts (MEFs) were maintained in the same basic medium, supplemented with non-essential amino acids, glutamine, Fungizone® and gentamicin (Invitrogen). HEK293 and Plat-E cells were transfected by calcium chloride transfection [57]. HEK293 cells stably transfected with shRNAs encoding pRSMX_PG vectors were selected using puromycin (Sigma). Analysis of endocytosis by flow cytometry was done at least two days after transfection. MEFs were infected by co-culture with filtered (0.2 µm filter) Plat-E supernatant, in the presence of Polybrene® (2 µg/mL). Transfection/infection efficiencies were verified by analysis of GFP percentage by flow cytometry (Accuri C6, Ann Arbor, MI, USA).

### DNA Constructs, Vectors and Mutagenesis

A human *PICALM* cDNA lacking both exon 17a [NGMHFPQY] and a portion of exon 13 [DPFSATV] was subcloned into the GFPN1 vector (Stratagene, La Jolla, CA, USA), downstream of the GFP gene. Full length PICALM and all point mutants included a hemagglutinin (HA) carboxy-terminal tag followed by a stop codon. PICALM truncation mutants were generated by PCR using oligonucleotides to introduce a 3′ TAA stop codon without a HA tag. All infections into MEFs for rescue assays were performed using human *PICALM* cDNA subcloned into the bicistronic MSCV-IRES-eGFP (MIE) retroviral vector [58]. Deletion *PICALM* constructs expressed in the MIE vector were generated by standard PCR protocols using oligonucleotides that hybridized to the appropriate regions of *PICALM* with flanking *XhoI* (5′ end) or *BamHI* (3′ end) restriction sites and a TAA stop codon at the 3′ end immediately after the indicated amino acid. All point mutations within the *PICALM* gene were made using the Stratagene Site Directed Mutagenesis kit (Agilent Technologies, Santa Clara, CA). The NPF mutant changed the indicated codons to encode AAA (5′-GCGGCCGCT-3′), incorporating a *NotI* site within the mutation site. NAAIRS mutagenesis [34], [59] was performed by replacing consecutive stretches of six amino acids with sequence encoding the amino acids NAAIRS (5′-AATGCAGCAATAAGATCT-3′), as well as a *BglII* site to facilitate screening. Primary MEFs were immortalized with the SV40 DNA tumor virus early region T/t antigen in the pBABE vector (courtesy of Dr. Corinne Linardic, Duke University).

*Picalm* shRNA were expressed using the GFP-encoding pRSMX_PG retroviral vector [60] (kindly provided by L. Staudt, NIH). The control shRNA directed against luciferase (5′-GTGGATTTCGAGTCGTCTTTAAT-3′), *Picalm* shRNA4 (5′-GCCTTAATGTTGACTTTGAAT-3′), and *Picalm* shRNA5 (5′-GCAGCATACAATGAAGGAATT-3′) were cloned into *HindIII* and *BglII* sites.

### Antibodies and Reagents

The following reagents were used: PE-conjugated-mouse-anti-CD71 (eBioscience, San Diego, CA, USA), Alexa Fluor® 633 conjugated transferrin (Invitrogen), goat-anti-PICALM antibody (C-18, Santa Cruz Biotechnologies, Santa Cruz, CA, USA), mouse-anti-clathrin heavy chain (TD.1, Sigma-Aldrich, St. Louis, MO, USA), rabbit anti- PICALM antibody (Sigma), mouse-anti-actin antibody (AC-40, Sigma), rabbit-anti-actin antibody (Sigma), mouse-anti TfR antibody (Invitrogen), Phen Green SK diacetate (Invitrogen), deferoxamine mesylate salt (DFO, Sigma), ammonium iron (III) citrate (FAC, Sigma), and rabbit-anti-GFP antibody (Stratagene).

### Cell Surface Labeling of Transferrin Receptor and Endocytosis of Transferrin/transferrin Receptor

HEK293 cells or MEFs growing exponentially were serum starved for 2 hours before being trypsinized briefly with 0.05% trypsin/EDTA (Invitrogen) until only 30–40% of the cells were lifting off the plate to minimize cleaving of surface TfR. Trypsinization was stopped with serum-containing medium and cells were washed with cold PBS and resuspended in 1% bovine serum albumin. Samples to be analyzed with anti-CD71 antibody were blocked using rat IgG (1 µg/mL). Alexa Fluor® 633-conjugated transferrin (50 µg/mL) or PE-conjugated anti-CD71 antibody was added to the cells, and the samples were incubated on ice for 1 hour in the dark. Cells were washed twice with cold PBS and analyzed by flow cytometry (C6, Accuri) to measure surface TfR levels, or further processed to measure rate of TfR endocytosis. For endocytosis assays, each sample was divided into multiple tubes that were incubated in a 25°C water bath for the indicated times. Endocytosis assays were conducted at 25°C instead of 37°C to slow down the process in order to more accurately record early time points. TfR internalization was stopped by adding ice-cold acid wash buffer (0.5 M NaCl/0.2 M acetic acid) to strip residual surface transferrin or anti-CD71 antibody. Cells were then washed twice with cold PBS and analyzed by flow cytometry. The rate of TfR endocytosis was determined from the slope of internalization measured between 3 and 5 minute time points and was normalized to the control vector. The GFP-positive gated cell population was analyzed for CD71 expression or bound transferrin. All experiments contained empty vector controls for comparison.

### Western Blots

Western blots were performed according to standard protocols. Proteins were detected by either alkaline phosphatase chemiluminescence methods or by quantitative immunoblot methods using an Odyssey infrared fluorescence imaging system (Li-Cor Biosciences, Lincoln, NB, USA).

### *Picalm* ^fit1^ Mice

ENU saturation mutagenesis studies originally resulted in the identification of five independent *fit1* mutations of varying severity [40]. Characterization of hematopoietic and iron deficiency phenotypes has previously been described in mice bearing an allele of intermediate severity, *fit1* ^4R^ (formerly *fit1* ^4226SB^) [9]–[11]. We chose to focus on the most severe allele, *fit1* ^5R^ (formerly *fit1* ^4397SB^), which results in a severely truncated and nonfunctional PICALM protein [7], [8]. *Picalm* ^+^/*Picalm* ^fit1–5R^ (hereafter, *fit1* ^+/−^) mice were obtained from Oak Ridge National Laboratory (Oak Ridge, TN, USA) and housed under normal conditions in accordance with the Duke University Institutional Animal Care and Use Committee.

### Mouse Embryonic Fibroblast Preparation

Heterozygous *Picalm* ^+^/*Picalm* ^fit1–5R^ mice were bred to generate homozygous mutant (*Picalm* ^fit1–5R^/*Picalm* ^fit1–5R^; herafter referred to as *Picalm* ^NULL^ or Null) and WT (*Picalm* ^+^/*Picalm* ^+^) embryos, which were harvested at 14.5 dpc. After removal of cranial (used for genotype screening) and liver tissue, the embryo was minced using a sterile razor blade. Following trypsinization, cells were serially replated several times. Within a week following extraction, primary MEFs were either analyzed or immortalized using SV40 T/t antigen. In all experiments, at least one *Picalm* ^NULL^ (Null) and one WT cell line was generated from the same pregnancy, and kept under identical conditions. The following immortalized MEFs were established from littermates from three separate pregnancies: pregnancy #1–3T (Null) and 7T (WT); pregnancy #2–20T (WT), 24T (Null), and 29T (Null); and pregnancy #3–31T (Null) and 33T (WT).

### Co-immunoprecipitation Assay

HEK293T cells were harvested 48 hours after transfection, and protein concentration was quantified by Bradford Assay (Thermo). One µL of anti-GFP antibody was added to 500 ng of total protein and incubated overnight at 4°C. Protein-G-Sepharose (GE Life Sciences) was added to the immunoprecipitates for 2 hr at 4°C. After three washes with lysis buffer, immunoprecipitates were analyzed by western blotting.

### Quantitative RT-PCR Analysis

Total RNA was isolated from MEFs using RNeasy Mini Kit (Qiagen, Valencia, CA, USA). Up to 1 µg of total RNA was treated with RNase-free DNase I and reverse transcribed using SuperScript II (Invitrogen) according to the manufacturer’s protocol. The 20 µL RT reaction was then diluted with 100 µL RNase-free H_2_O. One µL of diluted cDNA was used as template for quantitative PCR amplification using iQ Multiplex Powermix (Bio-Rad, Hercules, CA, USA) for 40 cycles according to the manufacturer’s protocol. Amplification data was collected using the iQ5 Optical System (Bio-Rad). The expression level of transferrin receptor transcript was quantified relative to the expression levels of the endogenous housekeeping genes, GAPDH (Sense: 5′-CCTGGAGAAACCTGCCAAGTATG-3′; Antisense: 5′-AGAGTGGGAGTTGCTGTTGAAGTC-3′) and β2M (Sense: 5′-ACCGGCCTGTATGCTATCCAGAAA-3; Antisense: 5′-GGTGAATTCAGTGTGAGCCAGGAT-3′) by the comparative threshold cycle (C_T_) method [61]. For transferrin receptor, the sense primer was 5′-TCATGAGGGAAATCAATGATCGTA-3′, and the antisense primer was 5′-GCCCCAGAAGATATGTCGGAA-3′.

### Intracellular Iron Measurement

The relative amount of intracellular iron was measured in MEFs using two separate approaches. First, total cellular iron content was determined by plating 10 million cells in 5 × 150 cm^2^ flasks the day prior to harvesting. On the following day, cells were trypsinized, washed, counted, and pelleted. Cell pellets were treated with nitric acid and analyzed by mass spectroscopy at the Soil Lab at North Carolina State University (Raleigh, NC). Iron concentration was normalized to represent the value per cell. Second, the labile iron pool was measured indirectly using the fluorophor Phen Green SK (PGSK – Invitrogen). All MEFs were plated at the same density the day before analysis. A total of 100,000 trypsinized cells were aliquoted in duplicates or triplicates, and 10 µM PGSK was added to each sample in the same final volume. Cells were incubated at 37°C for 30 minutes, pelleted, washed three times with HBSS, and analyzed by flow cytometry. Samples were run without PGSK and background fluorescence was subtracted from the PGSK fluorescence.

### Iron Supplementation and Chelation

For iron supplementation studies, non-immortalized MEFs were plated at a concentration of 50,000 cells/well in a 12 well plate with or without 50 µM ferric ammonium citrate (FAC) added both initially and on day 2 after plating. Cell proliferation was measured by counting viable cells following trypsinization at days two and five by flow cytometry.

For iron chelation experiments, immortalized MEFs were plated at a concentration of 25,000 cells/well in a 12 well plate with or without 2.5 µM deferoxamine (DFO) added on day 0 and day 2 after plating. Viable cells were counted at days two and four.

### Statistical Analysis and Preparation of Figures

We used the Wilcoxon rank-sum (Mann-Whitney) test to compare observations among groups, unless otherwise indicated. P values <0.05 were considered to be statistically significant. Data analysis and presentation were performed using Microsoft Excel and Stata 12 (College Station, TX). Figures were assembled in Adobe Photoshop.

## Supporting Information

Figure S1 **PICALM carboxy-terminal residues are required for inhibition of Tf endocytosis and clathrin binding.** **(A)** TfR internalization in HEK293 cells transiently transfected with PICALM deletion constructs is shown relative to empty vector control (GFP; upper panel). N_exp_  = 4. *p<0.002 compared with GFP vector. Lower panel shows corresponding Western blot of proteins co-immunoprecipitated using an anti-GFP antibody followed by immunoblotting with anti-CHC antibody. **(B)** TfR internalization in HEK293 cells transiently transfected with PICALM NAAIRS mutagenesis constructs is shown relative to empty vector control (GFP; upper panel). Point mutants are designated by the amino acids targeted by NAAIRS mutagenesis (e.g. aa 584–589 PTTAWN were mutated to NAAIRS). N_exp_  = 4. *p<0.002 compared with GFP vector. Lower panel shows corresponding Western blot of proteins co-immunoprecipitated using an anti-GFP antibody followed by immunoblotting with anti-CHC antibody. PICALM band (lane 1) was originally at the far right end of the gel and was moved to the left for clarity. **(C)** Schematic diagram showing location of NAAIRS mutants in PICALM C-terminus. Lower panel illustrates amino acid sequence of C-terminal PICALM, with numbers above sequence corresponding to PICALM amino acids 583–652. Streteches of six consecutive amino acids that were mutated to NAAIRS (asparagiNe-Alanine-Alanine-Isoleucine-aRginine-Serine) are indicated by square brackets above or below the sequence; these residues correspond to those shown in Figure S2B.
(TIF)

Figure S2 **Characterization of** ***PICALM-deficient*** **cell lines.** **(A)** *Picalm* PCR (upper) and immunoblot with anti-PICALM antibody (lower) confirm the absence of *Picalm* or PICALM expression in 4 *Picalm* ^NULL^ MEF lines (3T, 24T, 29T, 31T) compared with MEF lines derived from wildtype littermates (7T, 20T, 33T). Native PICALM protein typically appears as a doublet on immunoblots, with variable intensity of upper and lower bands. **(B)** Immunoblot of PICALM and β-actin protein in WT MEFs infected with shRNA vectors to knock down *Picalm* expression (shRNA4, shRNA5), control shRNA, or uninfected WT MEFs. **(C)** Immunoblot of native PICALM and β-actin in HEK293 cells stably transfected with shRNA4 or shRNA Control constructs, demonstrating knockdown of PICALM in comparison with β-actin. **(D)** Surface TfR expression in shRNA transduced HEK293 cells. N_exp_  = 7. *p<0.001 compared with shRNA Control. **(E)** TfR internalization in HEK293 cells that express shRNA4 or shRNA Control retroviruses. N_exp_  =  5. *p  = 0.001 at 3 min, p = 0.006 at 6 min compared with shRNA Control.
(TIF)

Figure S3 **Retroviral expression of PICALM in four independently derived** ***Picalm*** **^NULL^ MEF cell lines rescues TfR surface expression and endocytosis. (A)** Kinetics of TfR internalization in independently derived immortalized *Picalm* ^NULL^ lines (3T, 24T, 29T, 31T). Percentage of surface TfR internalized is shown for untransfected (Null), empty vector transfected (Control) and *PICALM*-transfected MEFs. (B) Relative surface TfR expression in four immortalized MEF *PICALM-deficient* MEF lines (3T, 24T, 29T, and 31T) derived from three different pregnancies. Surface TfR levels of empty vector transfected (Control) and *PICALM*-transfected MEFs are shown relative to untransfected (None) MEFs.
(TIF)
