# Supplementary figures and images for: The PICALM Protein Plays a Key Role in Iron Homeostasis and Cell Proliferation
Source: PLoS One. 2012 Aug 30;7(8):e44252. doi: 10.1371/journal.pone.0044252 (PMC3431333; doi:10.1371/journal.pone.0044252)

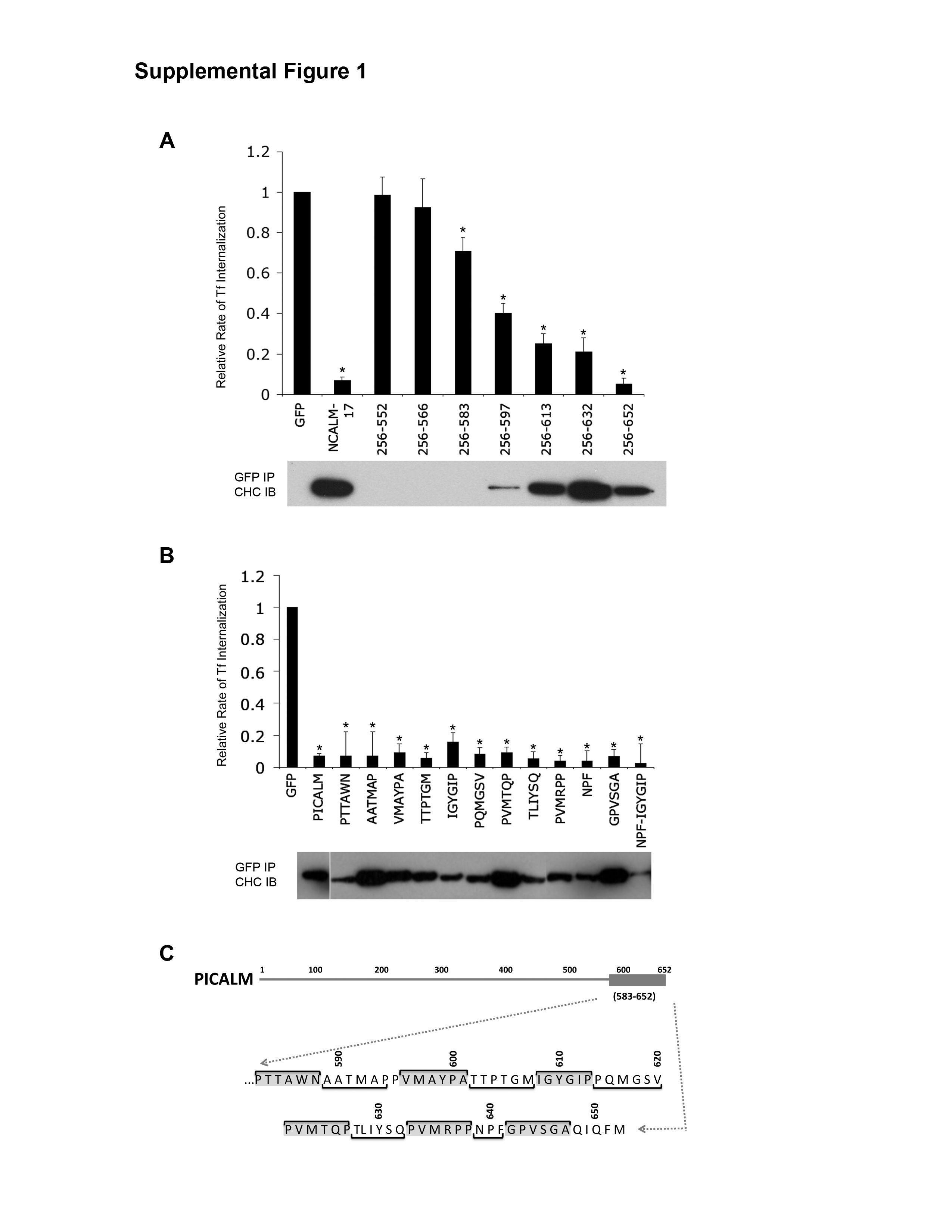

Supplement: Figure S1 — PICALM carboxy-terminal residues are required for inhibition of Tf endocytosis and clathrin binding. (A) TfR internalization in HEK293 cells transiently transfected with PICALM deletion constructs is shown relative to empty vector control (GFP; upper panel). Nexp = 4. *p<0.002 compared with GFP vector. Lower panel shows corresponding Western blot of proteins co-immunoprecipitated using an anti-GFP antibody followed by immunoblotting with anti-CHC antibody. (B) TfR internalization in HEK293 cells transiently transfected with PICALM NAAIRS mutagenesis constructs is shown relative to empty vector control (GFP; upper panel). Point mutants are designated by the amino acids targeted by NAAIRS mutagenesis (e.g. aa 584–589 PTTAWN were mutated to NAAIRS). Nexp = 4. *p<0.002 compared with GFP vector. Lower panel shows corresponding Western blot of proteins co-immunoprecipitated using an anti-GFP antibody followed by immunoblotting with anti-CHC antibody. PICALM band (lane 1) was originally at the far right end of the gel and was moved to the left for clarity. (C) Schematic diagram showing location of NAAIRS mutants in PICALM C-terminus. Lower panel illustrates amino acid sequence of C-terminal PICALM, with numbers above sequence corresponding to PICALM amino acids 583–652. Streteches of six consecutive amino acids that were mutated to NAAIRS (asparagiNe-Alanine-Alanine-Isoleucine-aRginine-Serine) are indicated by square brackets above or below the sequence; these residues correspond to those shown in Figure S2B. (TIF) [file pone.0044252.s001.tif]

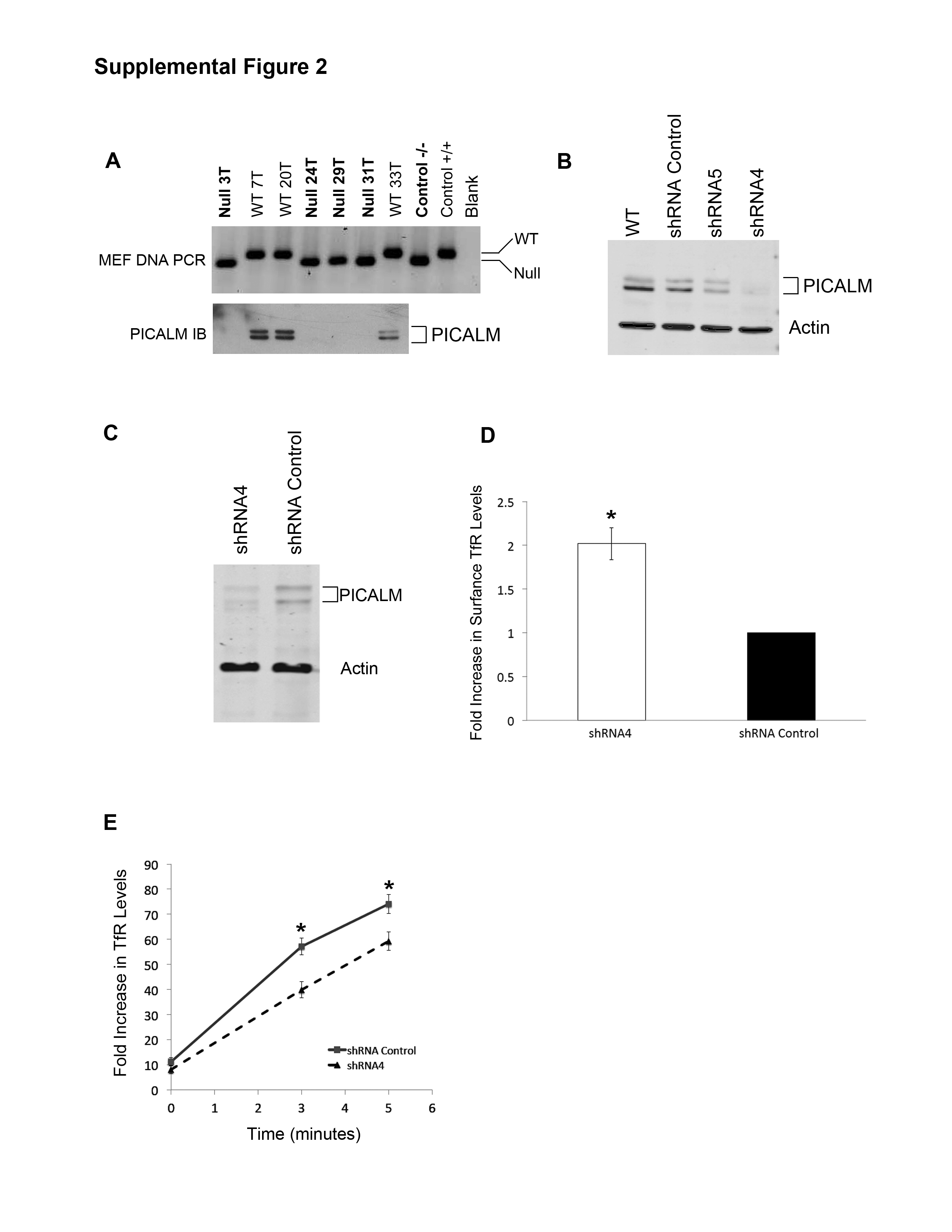

Supplement: Figure S2 — Characterization of PICALM-deficient cell lines. (A) Picalm PCR (upper) and immunoblot with anti-PICALM antibody (lower) confirm the absence of Picalm or PICALM expression in 4 Picalm NULL MEF lines (3T, 24T, 29T, 31T) compared with MEF lines derived from wildtype littermates (7T, 20T, 33T). Native PICALM protein typically appears as a doublet on immunoblots, with variable intensity of upper and lower bands. (B) Immunoblot of PICALM and β-actin protein in WT MEFs infected with shRNA vectors to knock down Picalm expression (shRNA4, shRNA5), control shRNA, or uninfected WT MEFs. (C) Immunoblot of native PICALM and β-actin in HEK293 cells stably transfected with shRNA4 or shRNA Control constructs, demonstrating knockdown of PICALM in comparison with β-actin. (D) Surface TfR expression in shRNA transduced HEK293 cells. Nexp = 7. *p<0.001 compared with shRNA Control. (E) TfR internalization in HEK293 cells that express shRNA4 or shRNA Control retroviruses. Nexp = 5. *p = 0.001 at 3 min, p = 0.006 at 6 min compared with shRNA Control. (TIF) [file pone.0044252.s002.tif]

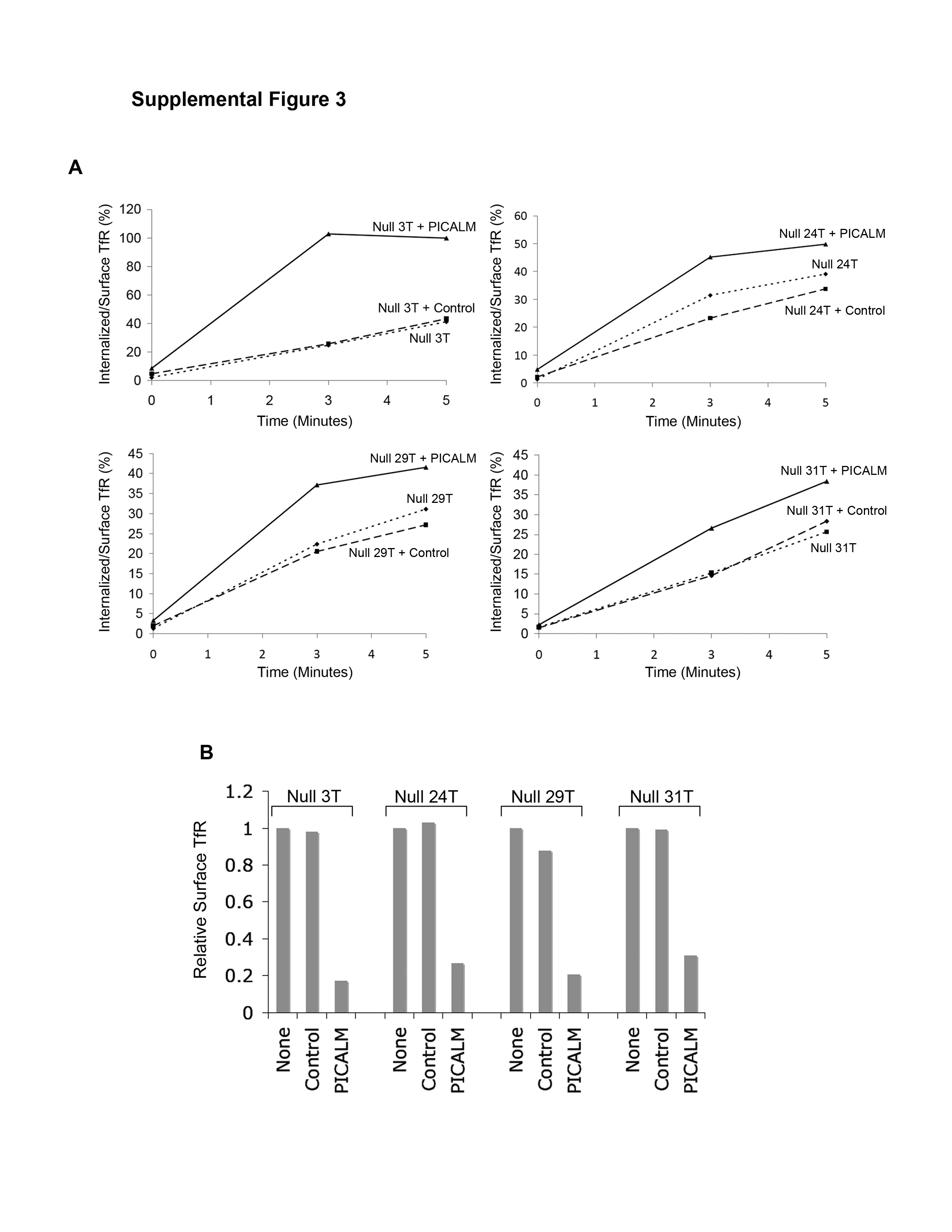

Supplement: Figure S3 — Retroviral expression of PICALM in four independently derived Picalm NULL MEF cell lines rescues TfR surface expression and endocytosis. (A) Kinetics of TfR internalization in independently derived immortalized Picalm NULL lines (3T, 24T, 29T, 31T). Percentage of surface TfR internalized is shown for untransfected (Null), empty vector transfected (Control) and PICALM-transfected MEFs. (B) Relative surface TfR expression in four immortalized MEF PICALM-deficient MEF lines (3T, 24T, 29T, and 31T) derived from three different pregnancies. Surface TfR levels of empty vector transfected (Control) and PICALM-transfected MEFs are shown relative to untransfected (None) MEFs. (TIF) [file pone.0044252.s003.tif]
